# Diagnosis and Treatment for Mild Cognitive Impairment: A Systematic Review of Clinical Practice Guidelines and Consensus Statements

**DOI:** 10.3389/fneur.2021.719849

**Published:** 2021-10-12

**Authors:** Ya-Xin Chen, Ning Liang, Xiao-Ling Li, Si-Hong Yang, Yan-Ping Wang, Nan-Nan Shi

**Affiliations:** ^1^Institute of Basic Research in Clinical Medicine, China Academy of Chinese Medical Sciences, Beijing, China; ^2^First School of Clinical Medicine, Shaanxi University of Chinese Medicine, Shaanxi, China

**Keywords:** diagnosis, guidelines, mild cognitive impairment, therapeutic, systematic review

## Abstract

**Background:** Mild cognitive impairment (MCI) is an important stage between the normal cognitive decline of aging and dementia. The aim of this study was to compare and harmonize the recommendations for the diagnosis and treatment of MCI based on current clinical practice guidelines.

**Methods:** We searched the PubMed, EMBASE, China National Knowledge Infrastructure, Wanfang Database, Chinese Science and Technology Periodical Database, and Chinese Biological Medicine Database from their inception date to April 24, 2021 to identify all published guidelines on MCI. The qualities of the eligible guidelines were appraised by two reviewers using the Appraisal of Guidelines for Research and Evaluation II instrument.

**Results:** Thirteen guidance documents (four guidelines and nine consensus statements) with specific recommendations were included. Nine guidelines and consensus statements covered the screening and diagnosis of MCI. The evaluation of the documents showed that neuropsychological testing and biomarker assessments were the most common recommendations for the diagnosis of MCI. Nine of the 13 guidance documents covered the treatment and management of MCI. The recommendations for the treatment and management were classified into four categories, namely: intervention for risk reduction, pharmacologic interventions, non-pharmacologic interventions, and counseling. Regarding pharmacological interventions, three guidelines recommend no pharmacologic intervention. The use of cholinesterase inhibitors for MCI is contraindicated in three guidance documents, whereas one proposes that cholinesterase inhibitors and memantine should be deprescribed. EHb761^®^, Chinese herbal decoctions, and Chinese traditional patent medicine are recommended in two documents. A total of seven guidance documents recommend non-pharmacological interventions, including physical activity interventions, cognitive interventions, dietary and nutritional interventions, and acupuncture.

**Conclusion:** An updated search for possible evidence on the diagnosis and treatment of MCI is needed. Potentially effective diagnoses and treatments, either conventional or complementary, and alternative therapies should be highly valued and addressed in correlation with the supporting evidence.

## Introduction

Mild cognitive impairment (MCI) is regarded as the transitional period between the normal cognitive decline of healthy aging and dementia (1). It is categorized into amnestic and non-amnestic subtypes (2). MCI is one of the most common diseases of the elderly, and it increases the risk of developing dementia (1). According to recent studies, the prevalence of MCI among elderly people in China is 15.5% (3), 13.11% in Greece (4), and 26.06% in South India (5). Moreover, it has been reported that 6–15% of patients with MCI develop Alzheimer's disease (AD) annually, and each patient with AD would spend $40,000 each year (6). The high conversion rate from MCI to AD places a large financial burden on public health. Thus, it is critical to pay attention to the diagnosis and treatment of MCI.

The first Mayo diagnostic criteria for MCI proposed by Petersen et al. (7) was mainly based on memory problems. The criteria comprised of memory complaint, normal activities of daily living, normal general cognitive function, abnormal memory for the age of the patient, and no signs of dementia (7). However, considering the non-memory problems that can also result in cognitive impairment, Petersen amended the Mayo criteria by adding non-memory criteria (8). Multiple tests, such as the mini-mental state examination (MMSE), the Montreal Cognitive Assessment (MoCA), the Free and Cued Selective Reminding Test (FCSRT), the California Verbal Learning Test, and the Boston Naming Test, have been proposed for screening for MCI. However, no specific accepted test and cutoff score have been recommended for the diagnosis of MCI. This lack of specific standardized tests of assessment cut-off points may influence the accuracy of the diagnosis (8–10). Furthermore, owing to the complexity of risk factors and the lack of clarity in the diagnosis of MCI, it is also difficult to provide specific treatment for patients. Currently, there are no accepted drugs for the treatment MCI (11). However, new management measures for MCI, such as cognitive interventions, exercise, and herbal medicine, are continuously introduced in clinical practice guidelines (12, 13).

Guidance documents, such as clinical practice guidelines and consensus statements, are developed using a systematic method to provide guidance and recommendations for clinicians (14). Guidance documents may focus on different topics, such as screening, diagnosis, or treatment (14). There are several published guidelines and consensus statements for MCI. However, discrepancies exist between the guidelines because of variations in topics and the time-sensitive nature of some of the evidence. Another reason for the variations between the guidelines may be that the guidelines from different countries are based on different sources and qualities of evidence. Hence, it is necessary to summarize and compare MCI recommendations to enable the clinicians to make more thoughtful clinical decisions and to encourage MCI guideline developers to consider evidence comprehensively. The discovery and evaluation of controversial recommendations is an advantage for future research. Thus, the aim of this study was to systematically review, compare, and harmonize the recommendations for the diagnosis and treatment of MCI in the current clinical practice guidelines.

## Methods

### Eligibility Criteria

Clinical practice guidelines and expert consensus were included if they satisfied the following criteria:

- with specific recommendations
- focus on diagnosis and/or treatment of patients with MCI
- not intended for MCI general practice, education, training, certification, and research
- not protocols, abstracts, editorial comments, overview/review articles, and systematic reviews.

### Search and Selection

The PubMed, EMBASE, China National Knowledge Infrastructure, Wanfang Database, Chinese Science and Technology Periodical Database, and Chinese Biological Medicine Database were systematically searched from their inception date to April 24, 2021. The keywords were Cognitive Dysfunction [MeSH] OR MCI [Title/Abstract] OR cognitive impairment [Title/Abstract] OR cognitive disorder* [Title/Abstract] OR cognitive dysfunction* [Title/Abstract] OR neurocognitive disorder* [Title/Abstract] OR cognitive decline* [Title/Abstract] OR mental deterioration* [Title/Abstract] AND Guideline [MeSH] OR expert consensus [Title/Abstract] OR recommendation statement [Title/Abstract]. The keywords mentioned above were adapted and searched in Chinese databases by using corresponding Chinese words; the search strategies are presented in Supplementary Table 1. Two authors (YC and NL) independently selected the clinical practice guidelines and consensus statements. The websites including Google Scholar, National Institute for Health and Care Excellence, Scottish Intercollegiate Guidelines Network, Guidelines International Network, and World Health Organization were also searched for gray literature. The languages were limited to English and Chinese.

### Basic Characteristics of the Included Documents and Extraction of Recommendations

The reviewers (NL and YC) extracted the basic characteristics and recommendation content of the included guidelines. The basic characteristics included guideline development information (development group, country, year, and number of guideline versions), guideline scope and content (target population, diagnosis, and treatment), evidence support (systematic search and number of references), grading systems of recommendations (level of evidence and strength of recommendations), and conflict of interest (type of funding).

The included guidelines were reviewed, and the recommendations were extracted. Information on the level of evidence and strength of recommendations for diagnosis (or screening) and treatment (or management) were extracted separately. For guidelines with more than one version but which were developed by the same organization or group, the recommendations on the diagnosis and treatment of MCI were extracted from the most updated version.

### AGREE II Assessment

The qualities of the eligible guidelines were independently appraised by two reviewers (YC and XL) using the Appraisal of Guidelines for Research and Evaluation (AGREE) II instrument. The results of the assessment were checked by the third author (NL). The AGREE II instrument comprises 23 items organized in six quality domains: scope and purpose, stakeholder involvement, rigor of development, clarity of presentation, applicability, and editorial independence. Each of the 23 items is rated on a seven-point scale. A score of 1 indicates “strongly disagree,” whereas a score of 7 represents “strongly agree.” The score of each domain is calculated based on the scores of its specific items (15, 16). Based on the methodology outlined in a previously published paper, we divided the domain scores into three groups: high quality (67–100%), sufficient quality (33–67%), and low quality (0–33%) (17, 18).

## Results

### Selection of Guidelines

**Figure 1:**
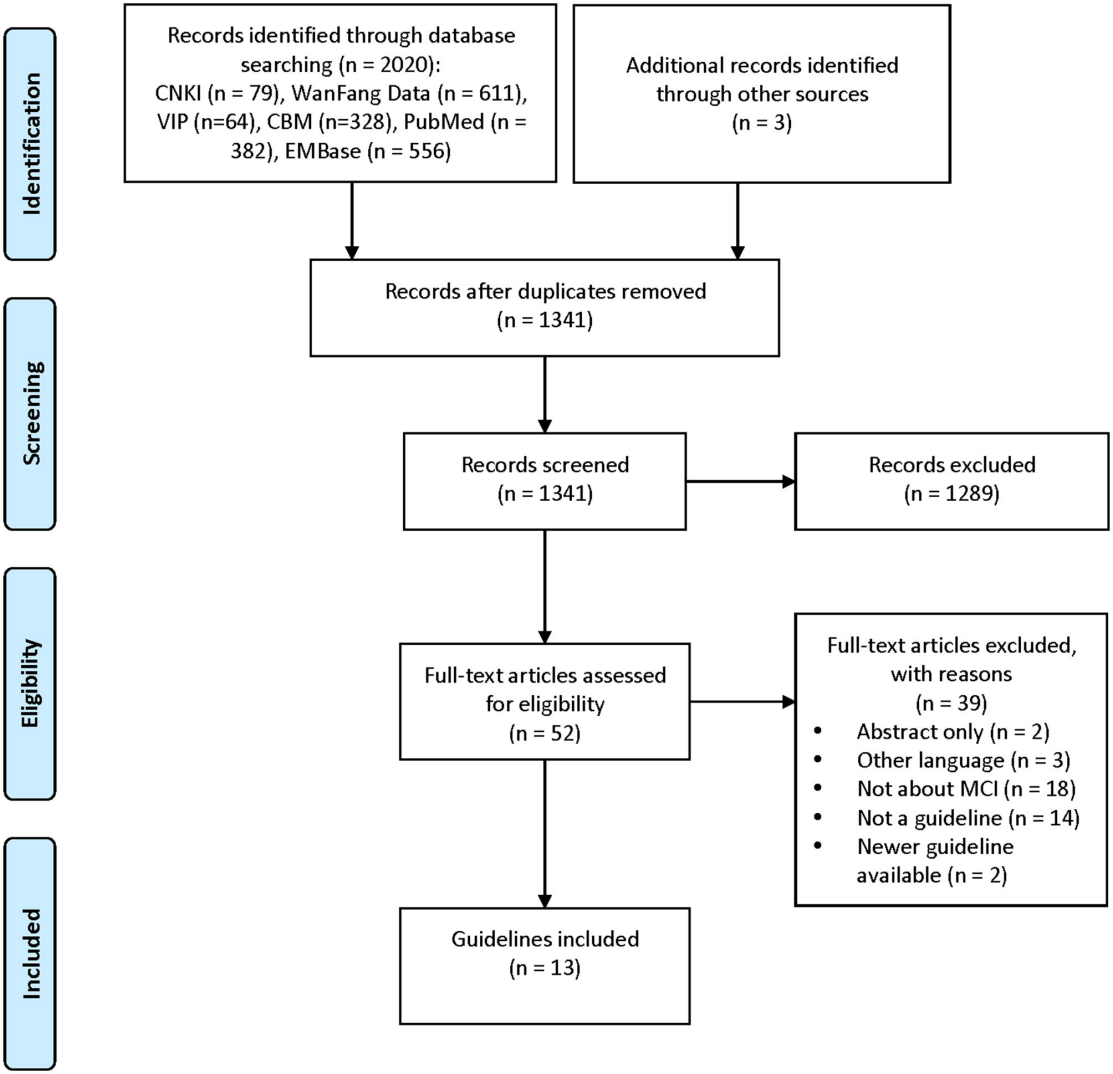
Screening chart of this study.

A total of 13 guidance documents with specific recommendations were eligible, including four guidelines (10, 12, 19, 20) and nine consensus statements (13, 21–28). A detailed flow chart of the search and selection is presented in Figure 1.

Two documents (12, 24) were developed in the United States, two (21, 28) in the United Kingdom, four (10, 19, 25, 27) in China, one (26) in Canada, one (13) in Singapore, one (20) in Switzerland, and two (22, 23) in Europe. Five of the documents had previous versions (10, 12, 13, 21, 26). Eleven documents (10, 12, 13, 20–26, 28) are directed at MCI populations and three documents (19, 26, 27) are directed at vascular mild cognitive impairment (VaMCI) populations. Recommendations for screening and diagnosis are included in nine documents (10, 12, 13, 19, 22–24, 26, 28), whereas recommendations for treatment and management are included in nine documents (10, 12, 13, 20, 21, 24–27). Nine documents state that the guideline/statement was developed using a systematic search strategy (10, 12, 19–24, 26). The detailed characteristics of the eligible guidelines and consensus statements are presented in Table 1. A summary of the grading systems used in the included guidelines and consensus statements is presented in Supplementary Table 2.

**Table 1:** Guidelines and consensus statement included in this paper.

**Guideline**	**Institution/Group**	**Country,** **territory or** **area of** **development**	**Year**	**Previous** **version**	**Target**	**Screening** **and diagnosis**	**Treatment and** **management**	**Number of** **references**	**Evidence** **based**	**Grading of** **evidence**	**Funding** **type**
Tian et al. (19)	Alzheimer's and Related Professional Committees of China Association of Elderly Health Care	China	2016	No	VaMCI	Yes	No	80	Yes	Yes	N/A
Brien et al. (21)	British Association for Psychopharmacology (BAP)	UK	2017	2 (2006, 2011)	MCI	No	Yes	146	Yes	Yes	I
Petersen et al. (12)	American Academy of Neurology (AAN)	USA	2018	1 (2001)	MCI	Yes	Yes	103	Yes	Yes	I
Jia et al. (10)	Cognitive Disorders Professional Committee of Neurologist Branch of Chinese Medical Doctor Association	China	2018	2 (2010, 2015)	MCI	Yes	Yes	22	Yes	Yes	G
Jia et al. (25)	Cognitive Disorders Professional Committee of Neurologist Branch of Chinese Medical Doctor Association	China	2019	No	MCI	No	Yes	24	N/A	Yes	G
Cummings et al. (24)	N/A	USA	2019	No	MCI	Yes	Yes	111	Yes	No	I
WHO (20)	WHO	Switzerland	2019	No	MCI	No	Yes	N/A	Yes	Yes	G
Zhou et al. (27)	State Administration of Traditional Chinese Medicine; Chinese Society of Traditional Chinese Medicine	China	2020	No	VaMCI	Yes	Yes	40	N/A	Yes	G
Ismail and Richard (26)	Canadian Consensus Conferences on the Diagnosis and Treatment of Dementia (CCCDTD)	Canada	2020	4 (1989, 1998, 2006, 2012)	MCI, VaMCI	No	Yes	123	Yes	Yes	I
Kandiah and Christopher (13)	Asian Clinical Expert Group on Neurocognitive Disorders (ASCEND)	Singapore	2021	1 (2019)	MCI	Yes	Yes	115	Yes	Yes	P
Dunne et al. (28)	N/A	UK	2021	No	MCI	Yes	Yes	77	Yes	No	N/A
Herukka et al. (22)	N/A	European	2017	No	MCI	Yes	No	51	Yes	Yes	G; I
Nobili et al. (23)	European Association of Nuclear Medicine (EANM) and European Academy of Neurology (EAN)	European	2018	No	MCI	Yes	No	119	Yes	Yes	G; I; P

*I, institutional; G, governmental; P, pharmaceutical company; N/A, not applicable*.

### Quality Assessment

The scores for each domain of the AGREE II instrument are presented in Table 2. The guideline developed by the American Academy of Neurology (AAN) had the highest overall score in the six domains (12). Nine guidance documents (12, 13, 19–24, 26) had high scores in the “scope and purpose” domain (domain 1), whereas four (10, 25, 27, 28) had sufficient scores (domain 1: average, 68%; range, 44–78%). The scores for the “stakeholder involvement” (domain 2) were generally low; all documents scored below 67% in that domain. Eight documents (12, 13, 20, 21, 23, 26–28) had sufficient scores in domain 2, whereas five (10, 19, 22, 24, 25) had low scores (domain 2: average, 37%; range, 22–66%). Two documents (12, 20) had high quality in the “rigor of development” domain, four (13, 22, 23, 26) had sufficient quality, and the others had low quality (domain 3: average, 41%; range, 19–71%). The “clarity of presentation” domain (domain 4) had the highest scores among the six domains (domain 4: average, 74%; range, 47–86%). Ten of the 13 documents were rated as high quality in domain 4 (10, 12, 13, 19–21, 24–26, 28). Overall, “applicability” domain (domain 5) had the lowest scores among the six domains (domain 5: average, 29%; range, 6–48%). Six documents (12, 22–24, 26, 28) were rated as with sufficient quality in domain 5, whereas others were rated as with low quality. Five documents were of low quality in the “editorial independence” domain (domain 6: average 47%; range, 0–92%) (10, 19, 22, 25, 27), two (23, 26) were of sufficient quality, and six (12, 13, 20, 21, 24, 28) were of high quality.

**Table 2:** Assessment of guidelines and consensus statements by AGREE II.

	**Domain 1**	**Domain 2**	**Domain 3**	**Domain 4**	**Domain 5**	**Domain 6**	**Overall (%)**
**Guideline**	**Scope and** **purpose (%)**	**Stakeholder** **involvement (%)**	**Rigor of** **development (%)**	**Clarity of** **presentation (%)**	**Applicability** **(%)**	**Editorial** **independence (%)**	
Tian et al. (19)	69	25	29	72	6	0	50
Petersen et al. (12)	77	66	71	86	47	91	92
Brien et al. (21)	69	39	27	86	31	88	58
Jia et al. (10)	61	25	27	69	12	4	42
Jia et al. (25)	44	22	20	77	16	0	33
Cummings et al. (24)	72	33	32	78	35	83	58
WHO (20)	78	61	82	81	40	75	83
Zhou et al. (27)	66	47	19	63	18	0	25
Ismail and Richard (26)	67	50	53	86	42	54	67
Kandiah and Christopher (13)	67	25	45	81	27	71	58
Dunne et al. (28)	61	25	26	81	35	92	50
Herukka et al. (22)	78	25	53	47	48	13	50
Nobili et al. (23)	69	44	53	56	19	46	58
Average	68	37	41	74	29	47	

*Green color, 67–100 (high quality); yellow color, 34–66 (sufficient quality); red color, 0–33 (low quality) (18)*.

### Screening and Diagnosis

Nine guidelines and consensus statements covered the screening and diagnosis of MCI (10, 12, 13, 19, 24, 26, 28). Neuropsychological testing and biomarker assessments are the most recommended tests for the diagnosis of MCI. There was agreement between two guidance documents recommending that standard diagnostic criteria, such as the Vascular Behavioral and Cognitive Disorders Society criteria, Diagnostic and Statistical Manual of Mental Disorders 5, Vascular Impairment of Cognition Classification Consensus Study, or the American Heart Association consensus statement, should be used in the diagnostic process for VaMCI (19, 26). Two guidelines recommend that self-report from patients should not be solely relied on for clinical history but should be supplemented by reports from people familiar with the patient (10, 19). Three guidance documents indicate that clinicians should combine clinical history with neuropsychological testing in the diagnostic process (12, 24, 26). One guideline recommends making a diagnosis of MCI subtype (10). Two guidance documents recommend that clinicians identify the MCI risk factors that are potentially modifiable (12, 28). Seven documents (10, 12, 13, 19, 24, 26, 28) recommend the use of neuropsychological testing for screening and diagnosis. Three (10, 19, 26) of these seven documents recommend cognitive testing, including assessment using the Modified Mini-Mental State (3MS) examination, the MMSE, the Rowland Universal Dementia Assessment Scale (RUDAS), MoCA, Toronto Cognitive Assessment (TorCA), National Institute for Neurological Disorders and Stroke and Canadian Stroke Network (NINDS-CSN), and FCSRT; three (10, 13, 19) recommend testing activities of daily living and functional assessment, including assessment using the Activity of Daily Living Scale (ADL), Instrumental Activity Daily Living (IADL) scale, and Functional Activities Questionnaire (FAQ); and one document (10) recommends behavioral and psychological assessment. Three documents (12, 24, 28) do not recommend a specific diagnostic tool, and one guidance document does not recommend cognitive testing for screening asymptomatic adults (26). Three guidance documents (10, 19, 26) propose that a physical examination needs to be conducted for diagnosis and for the prediction of the progression to dementia. Two (10, 26) of these three documents recommend dual-task gait test, while one document recommends the olfactory function test and hearing test (10). Regarding diagnostic radiologic examinations, two documents (12, 19) recommend magnetic resonance imaging (MRI), and three documents (12, 19, 23) recommend positron emission tomography (PET). One guideline recommends the assessment of medial temporal lobe atrophy for the identification of hippocampal atrophy (19). Two guidelines recommend conducting blood tests to exclude other potential diseases (10, 19). Five guidance documents (10, 13, 22, 24, 28) recommend biomarker assessments to help confirm the diagnosis of MCI. Three (10, 22, 28) of these documents recommend cerebrospinal fluid biomarker tests (CSF tau protein and CSF β-amyloid 42), whereas another guideline (12) suggests that there is no accepted biomarker for a definite diagnosis. Three guidance documents recommend follow-up or monitoring the changes in the cognitive status of a patient with MCI (10, 12, 28). The details and levels of recommendations for screening and diagnosis are presented in Table 3 and Supplementary Table 3A.

**Table 3:** Summary of recommendations for screening and diagnosis.

**Recommendations**	**Tian et al. (19)**	**Herukka et al. (22)**	**Nobili et al. (23)**	**Petersen et al. (12)**	**Jia et al. (10)**	**Cummings et al. (24)**	**Kandiah and Christopher (13)**	**Dunne et al. (28)**	**Ismail and Richard (26)**
Use VAS-COG Society criteria, DSM5, vascular impairment of cognition Classification consensus study or the American Heart Association consensus statement for diagnosis of VaMCI	+	N/A	N/A	N/A	N/A	N/A	N/A	N/A	+
Clinical history cannot rely solely on the self-report from patients and should be supplemented by familiar people	+	N/A	N/A	N/A	+	N/A	N/A	N/A	N/A
Combining clinical history with neuropsychological testing when making diagnosis	N/A	N/A	N/A	+	N/A	+	N/A	N/A	+
Making a diagnosis of MCI subtype	N/A	N/A	N/A	N/A	+	N/A	N/A	N/A	N/A
Identifying risk factors that are potentially modifiable	N/A	N/A	N/A	+	N/A	N/A	N/A	+	N/A
Use of neuropsychological testing for screening and diagnosis	+	N/A	N/A	+	+	+	+	+	+
Cognitive testing	N/A	N/A	N/A	N/A	+	N/A	N/A	N/A	N/A
Cognitive testing for screening asymptomatic adults	N/A	N/A	N/A	N/A	N/A	N/A	N/A	N/A	-
3MS examination	N/A	N/A	N/A	N/A	N/A	N/A	N/A	N/A	+
MMSE	N/A	N/A	N/A	N/A	+	N/A	N/A	N/A	+
RUDAS	N/A	N/A	N/A	N/A	N/A	N/A	N/A	N/A	+
MoCA	N/A	N/A	N/A	N/A	+	N/A	N/A	N/A	+
TorCA	N/A	N/A	N/A	N/A	N/A	N/A	N/A	N/A	+
NINDS-CSN	+	N/A	N/A	N/A	N/A	N/A	N/A	N/A	N/A
FCSRT	N/A	N/A	N/A	N/A	+	N/A	N/A	N/A	N/A
Activities of daily living testing and functional assessment	+	N/A	N/A	N/A	+	N/A	+	N/A	N/A
ADL	N/A	N/A	N/A	N/A	N/A	N/A	+	N/A	N/A
IADL	+	N/A	N/A	N/A	+	N/A	N/A	N/A	N/A
FAQ	N/A	N/A	N/A	N/A	+	N/A	N/A	N/A	N/A
Behavioral and psychological assessment	N/A	N/A	N/A	N/A	+	N/A	N/A	N/A	N/A
Use of physical examination for diagnosis and predicting the progression to dementia	+	N/A	N/A	N/A	+	N/A	N/A	N/A	N/A
Dual-task gait test	N/A	N/A	N/A	N/A	+	N/A	N/A	N/A	+
Olfactory function test	N/A	N/A	N/A	N/A	+	N/A	N/A	N/A	N/A
Hearing test	N/A	N/A	N/A	N/A	+	N/A	N/A	N/A	N/A
Use of neuroimaging for diagnosis	+	N/A	N/A	N/A	N/A	N/A	N/A	N/A	N/A
Structural imaging	+	N/A	N/A	N/A	N/A	N/A	N/A	N/A	N/A
MRI	+	N/A	N/A	N/A	+	N/A	N/A	N/A	N/A
MTA scale for identifying hippocampal atrophy	+	N/A	N/A	N/A	N/A	N/A	N/A	N/A	N/A
Functional imaging	N/A	N/A	N/A	N/A	N/A	N/A	N/A	N/A	N/A
PET	+	N/A	+	N/A	+	N/A	N/A	N/A	N/A
Use of blood tests for excluding other diseases	+	N/A	N/A	N/A	+	N/A	N/A	N/A	N/A
Use of biomarker assessments for helping confirm diagnosis	N/A	+	N/A	-	+	+	+	+	N/A
CSF biomarker	N/A	+	N/A	N/A	+	N/A	N/A	+	N/A
Follow-up of MCI patients or monitoring the change in cognitive status	N/A	N/A	N/A	+	+	N/A	N/A	+	N/A

*VAS-COG, Vascular Behavioral and Cognitive Disorders; DSM5, Diagnostic and Statistical Manual of Mental Disorders; VaMCI, vascular mild cognitive impairment; MCI, mild cognitive impairment; 3MS, Modified Mini-Mental State; MMSE, Mini-Mental State Examination; RUDAS, the Rowland Universal Dementia Assessment Scale; MoCA, Montreal Cognitive Assessment; TorCA, Toronto Cognitive Assessment; NINDS-CSN, National Institute for Neurological Disorders and Stroke and Canadian Stroke Network; FCSRT, free and cured selective reminding test; MRI, magnetic resonance imaging; PET, positron emission tomography; CSF, cerebrospinal fluid; +, recommended for screening for MCI; –, not recommended for screening in MCI; N/A, not applicable; green, strong recommendation; yellow, moderate recommendation; red, week recommendation; N/A, not applicable*.

### Treatment and Management

Nine guidance documents covered recommendations for the treatment (10, 12, 13, 20, 21, 24–27). The recommendations for treatment and management were classified into four categories: intervention for risk reduction, pharmacologic interventions, non-pharmacologic interventions, and counseling. Regarding intervention for risk reduction, one guideline (20) recommends that patients reduce or cease harmful drinking, whereas another guideline (12) recommends discontinuing medications that can contribute to cognitive impairment. Seven guidance documents include recommendations for pharmacologic interventions (10, 12, 13, 21, 24, 26, 27). Three guidelines (10, 12, 24) indicate that there is no accepted drug for the treatment of MCI. Three guidance documents (12, 13, 21) contraindicate cholinesterase inhibitors for the treatment of MCI, whereas one (26) proposes that cholinesterase inhibitors and memantine should be deprescribed. One consensus statement proposes that EHb761^®^ can improve the symptoms of MCI (13). One consensus statement recommends Chinese herbal decoction (Guipi decoction, Heche Dazao pills, Huanshaodan, Ditan decoction, and Tongqiao Huoxue decoction) and Chinese traditional patent medicine (FuFangCongRongYiZhi capsule, Tianzhi granules, Yangxue Qingnao granule, and Huanshao capsule) (27). Regarding non-pharmacologic interventions, four guidance documents (12, 13, 20, 26) recommend physical activity interventions, including aerobic exercise; four (12, 13, 20, 25) recommend cognitive interventions; three (20, 24, 27) recommend dietary and nutritional interventions, including a Mediterranean-like diet, Souvenaid, and Chinese medicine dietary therapy; and one (27) recommends acupuncture. Regarding counseling, a guideline proposes that clinicians should discuss prognosis and long-term planning with the patients and their families (12). The details and levels of treatment recommendations are presented in Table 4 and Supplementary Table 3B.

**Table 4:** Summary of recommendations for treatment and management.

**Recommendations**	**Petersen** **et al. (12)**	**Brien** **et al. (21)**	**Jia et al.** **(10)**	**Jia et al.** **(25)**	**Cummings** **et al. (24)**	**Zhou** **et al. (27)**	**WHO** **(20)**	**Ismail and** **Richard (26)**	**Kandiah and** **Christopher (13)**
Interventions for risk reduction	N/A	N/A	N/A	N/A	N/A	N/A	+	N/A	N/A
Interventions for alcohol use disorder	N/A	N/A	N/A	N/A	N/A	N/A	+	N/A	N/A
Ceasing medications which can cause cognitive impairment	+	N/A	N/A	N/A	N/A	N/A	N/A	N/A	N/A
Pharmacologic interventions	N/A	N/A	N/A	N/A	N/A	N/A	N/A	N/A	N/A
No accepted drug	+	N/A	+	N/A	–	N/A	N/A	N/A	N/A
Cholinesterase inhibitors	–	–	N/A	N/A	N/A	N/A	N/A	N/A	–
Cholinesterase inhibitors and memantine should be deprescribed	N/A	N/A	N/A	N/A	N/A	N/A	N/A	+	N/A
EHb761^®^ for improving symptoms	N/A	N/A	N/A	N/A	N/A	N/A	N/A	N/A	+
Chinese herbal decoction	N/A	N/A	N/A	N/A	N/A	+	N/A	N/A	N/A
Chinese traditional patent medicine	N/A	N/A	N/A	N/A	N/A	+	N/A	N/A	N/A
Non-pharmacologic interventions	N/A	N/A	N/A	N/A	N/A	N/A	N/A	N/A	N/A
Physical activity interventions	+	N/A	N/A	N/A	N/A	N/A	+	+	+
Aerobic exercise	N/A	N/A	N/A	N/A	N/A	N/A	N/A	+	N/A
Cognitive interventions	+	N/A	N/A	+	N/A	N/A	+	N/A	+
Dietary and nutritional interventions	N/A	N/A	N/A	N/A	+	+	+	N/A	N/A
Mediterranean-like diet	N/A	N/A	N/A	N/A	N/A	N/A	+	N/A	N/A
Souvenaid	N/A	N/A	N/A	N/A	+	N/A	N/A	N/A	N/A
Chinese medicine dietary therapy	N/A	N/A	N/A	N/A	N/A	+	N/A	N/A	N/A
Acupuncture	N/A	N/A	N/A	N/A	N/A	+	N/A	N/A	N/A
Counseling									
Discussing prognosis and long-term planning topics with patients and families	+	N/A	N/A	N/A	N/A	N/A	N/A	N/A	N/A

*+, recommended for screening for MCI; –, not recommended for screening in MCI; green, strong recommendation; yellow, moderate recommendation; red, week recommendation; N/A, not applicable*.

## Discussion

### Key Findings

As MCI is a transitional stage between the cognitive decline of healthy aging and dementia, its management is critical and requires considerable attention. In this study, we systematically collated MCI guidelines and incorporated the recommendations for review after appraising the qualities of the guidelines. We included a total of 13 MCI guidance documents in this review. The assessment of the guidelines using the AGREE II tool showed that the AAN guideline has the highest methodological quality of all the included guidelines (12). We analyzed the consistency of the recommendations but found some variation among the MCI guidelines.

Some differences were found among the nine guidance documents that include diagnostic recommendations. Neuropsychological assessment is recommended in seven guidelines; however, we noted some inconsistency in the guidelines (10, 12, 13, 19, 24, 26, 28). Three guidance documents do not recommend specific tests (12, 24, 28), whereas others recommend different tests, including MMSE, 3MS examination, MoCA, TorCA, RUDAS, NINDS-CSN neuropsychological protocols, FCSRT, IADL scale, and FAQ. Among the varied tests mentioned above, NINDS-CSN neuropsychological protocols, FCSRT, ADL, IADL scale, and FAQ are focused on testing deficits in a single cognitive domain (10, 19), while the MMSE and MoCA tests, which are the most common cognitive screening instruments, are used to evaluate multiple cognitive domains (10, 19). Three guidance documents recommend the use of more comprehensive neuropsychological tools for the assessment of multiple cognitive domains in patients with MCI (10, 19, 26). One document (19) states that cognitive assessment should include both a comprehensive test and at least four single-domain tests, while another document (10) states that cognitive assessment should include either a comprehensive test or several single-domain tests. An inconsistency was found regarding whether comprehensive tests and single-domain tests should be used alone or in combination. Moreover, both guidance documents do not clarify which specific test should be used. When choosing which cognitive test to use, clinicians should consider the sensitivity, specificity, and time efficiency of the test (29). The guidelines indicate that the sensitivity of the MMSE is low when it is used alone (10, 26). A transversal study of 229 elderly participants in four medical centers demonstrated that the MMSE has lower sensitivity than the MoCA (30). Three systematic reviews also demonstrated that the sensitivity and specificity of the MoCA are superior to those of the MMSE (29, 31, 32). Hence, the MoCA may be better than the MMSE as a comprehensive neuropsychological screening tool. Moreover, we found that none of the guidance documents included in this review has details of a cutoff score for the diagnosis of MCI. However, a systematic review concluded that the best cutoff point for MoCA score is 24/25 (31). Another systematic review suggested that the influence of education should be considered when using cutoff values (33). In conclusion, there is still no commonly accepted cutoff score for the diagnosis of MCI. Future studies that involve a further evaluation of the diagnostic accuracy of MCI using different tests and clearly defined cutoff values are needed.

There is a controversial question regarding whether the measurement of biomarkers is effective for the detection of progression toward AD in patients with MCI. The biomarkers can be divided into two categories: imaging-based biomarkers and CSF-based biomarkers (34). The imaging-based biomarkers include MRI-based biomarkers and PET-based biomarkers. MRI-based biomarkers contain the hippocampus and entorhinal cortex; PET-based biomarkers mainly contain amyloid PET, tau PET, and fluorine-18-fluorodeoxyglucose (FDG)-PET; CSF-based biomarkers mainly include CSF tau protein, CSF t-tau protein, and CSF β-amyloid peptide (Aβ_1−42_) (35–37). In the present study, six guidance documents covered biomarker assessments; however, they proposed different recommendations (10, 12, 13, 22, 24, 28). Five documents (10, 13, 22, 24, 28) recommend that biomarker assessments may help confirm the diagnosis of MCI and identify prodromal AD in patients with MCI. One (10) of these documents indicates that the CSF tau protein and CSF β-amyloid 42 tests for amnestic MCI (aMCI) are advantageous for screening patients with AD. However, another guideline indicates that there is no accepted biomarker for predicting the progression of MCI (12). Although assessment of biomarker is not recommended in that guideline, the authors state that they have a positive view of the biomarker (12). Only one guidance document recommends using same the cutoff points for CSF biomarkers regardless of apolipoprotein E genotype (22). There are also some contraindications for the application of CSF biomarker test, such as increased intracranial pressure and coagulopathy (22). About imaging-based biomarkers, two documents give the recommendations about the use of FDG-PET to confirm the diagnosis of AD in MCI (19, 23). FDG-PET is also recommended to ascertain the diagnosis of FTLD and DLB in MCI (23). The cost benefit of imaging-based and CSF-based biomarkers is another issue worth considering. The Manchester consensus of MCI includes three of 11 recommendations regarding the use of biomarkers for early detection. However, the consensus indicates that the cost utility of biomarkers remains controversial (28).

Variations also exist in the nine documents that include recommendations for the treatment and management of MCI. Three documents do not recommend pharmacological interventions for the treatment of MCI (13, 27). However, EHb761^®^ is recommended in one consensus statement for the improvement of MCI symptoms (13). EHb761^®^ is an extract from the *Ginkgo biloba* plant. A systematic review, which include 21 trials with 2,608 patients, concluded that *G. biloba* may help improve the cognitive function and activities of daily living of patients with MCI (38). Another consensus statement recommends Chinese herbal decoctions and Chinese traditional patent medicine for the treatment of VaMCI (27). A 2-year trial demonstrated that traditional Chinese medicine, such as Bushen capsules, may improve the cognitive performance of patients with aMCI (39). Herb medicine might be a potential treatment for the improvement of MCI symptoms; however, more high-quality evidence is needed to confirm their efficacy. Four guidance documents (12, 13, 20, 26) recommend physical activity interventions for MCI. One (26) of these documents recommends a specific physical intervention, aerobic exercise. A systematic review of randomized control trials indicates that aerobic and resistance exercises could improve cognitive function (40). Some studies have shown that specific physical activity interventions, such as mind–body exercise, Tai Chi, functional task exercise, and yoga, may significantly improve the MCI symptoms (41–44). The specific physical activity interventions suitable for patients with MCI, the duration of the exercises, and strength of the exercises need to be researched in future studies.

Although guidance on follow-up or monitoring of the changes of MCI patients have been provided by three guidance documents, no detailed information on how to manage it can be determined. As it is worthwhile to clarify how often the patients with MCI should be monitored and what specific assessments the patients should do, this problem needs to be addressed in future guidelines.

In general, most guidelines and consensus statements (seven of 11) included in the present study were of sufficient and low quality. The domain with the lowest score was the “applicability” domain (domain 5). “Applicability” is a key measure of the implementation of a guideline in clinical practice (16). Applicability limitations, lack of description of facilitators and barriers, provision of additional tools, and potential resources may potentially affect health improvement.

### Strengths and Limitations

To the best of our knowledge, this is the first paper to summarize the published guideline recommendations for MCI. The main strength of this study is the comprehensive and systematic literature search that was conducted to identify guidance documents related to the diagnosis and treatment of MCI. The guidelines and consensus statements were independently appraised by two reviewers using AGREE II, and the finding on the methodological problems, mainly in the applicability domain, presented as lacking implementation strategies and related application resources, may help in the development of future MCI guidelines. The recommendations were summarized into key recommendations shown in tables, and the consistency of the recommendations and the difference between them were compared.

The limitation of this study is that, although a systematic search strategy was implemented, certain guidelines may have been missed because of language limitations. Moreover, there were no accepted cutoff points for the domain scores of AGREE II. In addition, the fact that we referred to a previous article for the grading of the domain scores might be a matter of dispute (17, 18).

## Conclusions

An updated search of possible evidence for the diagnosis and treatment of MCI is needed. Potentially effective diagnoses and treatments, either conventional or complementary, and alternative therapies should be highly valued and addressed in correlation with the supporting evidence. The AGREE II and Reporting Items for Practice Guidelines in Healthcare tools can be referred to when developing guidelines.

## Data Availability Statement

The original contributions presented in the study are included in the article/Supplementary Material, further inquiries can be directed to the corresponding author/s.

## Author Contributions

NL and Y-XC designed the study, developed the analysis plan, analyzed the data, and contributed to the writing of the article. X-LL and S-HY extracted the data and appraised the quality of eligible guidelines. Y-PW and N-NS revised the manuscript and polish the language. All authors contributed to the article and approved the submitted version.

## Funding

This work was supported by the National Key Research & Development Program of China (Grant Nos. 2019YFC1712000, 2019YFC1712003, and 2019YFC1712005).

## Conflict of Interest

The authors declare that the research was conducted in the absence of any commercial or financial relationships that could be construed as a potential conflict of interest.

## Publisher's Note

All claims expressed in this article are solely those of the authors and do not necessarily represent those of their affiliated organizations, or those of the publisher, the editors and the reviewers. Any product that may be evaluated in this article, or claim that may be made by its manufacturer, is not guaranteed or endorsed by the publisher.

## References

[1] Anderson ND. State of the science on mild cognitive impairment (MCI). CNS Spectr. (2019) 24:78–87. 10.1017/S1092852918001347 30651152

[2] Nicolini P Mari D Abbate C Inglese S Bertagnoli L Tomasini E . Autonomic function in amnestic and non-amnestic mild cognitive impairment: spectral heart rate variability analysis provides evidence for a brain-heart axis. Sci Rep. (2020) 10:11661. 10.1038/s41598-020-68131-x 32669640 PMC7363846

[3] Jia L Du Y Chu L Zhang Z Li F Lyu D . Prevalence, risk factors, and management of dementia and mild cognitive impairment in adults aged 60 years or older in China: a cross-sectional study. Lancet Public Health. (2020) 5:e661–71. 10.1016/S2468-2667(20)30185-7 33271079

[4] Vlachos GS Kosmidis MH Yannakoulia M Dardiotis E Hadjigeorgiou G Sakka P . Prevalence of mild cognitive impairment in the elderly population in Greece: results from the HELIAD study. Alzheimer Dis Assoc Disord. (2020) 34:156–62. 10.1097/WAD.0000000000000361 31913961

[5] Mohan D Iype T Varghese S Usha A Mohan M. A cross-sectional study to assess prevalence and factors associated with mild cognitive impairment among older adults in an urban area of Kerala, South India. BMJ Open. (2019) 9:e025473. 10.1136/bmjopen-2018-025473 30898818 PMC6475216

[6] Petersen RC Stevens JC Ganguli M Tangalos EG Cummings JL DeKosky ST. Practice parameter: early detection of dementia: mild cognitive impairment (an evidence-based review). Neurology. (2001) 56:1133–42. 10.1212/WNL.56.9.1133 11342677

[7] Petersen RC Smith GE Waring SC Ivnik RJ Tangalos EG Kokmen E. Mild cognitive impairment: clinical characterization and outcome. Arch Neurol. (1999) 56:303–8. 10.1001/archneur.56.3.303 10190820

[8] Petersen RC. Mild cognitive impairment as a diagnostic entity. J Intern Med. (2004) 256:183–94. 10.1111/j.1365-2796.2004.01388.x 15324362

[9] Albert MS DeKosky ST Dickson D Dubois B Feldman HH Fox NC . The diagnosis of mild cognitive impairment due to Alzheimer's disease: recommendations from the National Institute on Aging-Alzheimer's Association workgroups on diagnostic guidelines for Alzheimer's disease. Alzheimers Dement. (2011) 7:270–9. 10.1016/j.jalz.2011.03.008 21514249 PMC3312027

[10] Jia J Writing Group of Chinese Guidelines for Diagnosis and Treatment of Dementia and Cognitive Impairment, Chinese Medical Doctor Association Neurologist Branch Cognitive Disorders Professional, Committee. Chinese guidelines for diagnosis and treatment of dementia and cognitive impairment in 2018 (five): diagnosis and treatment of mild cognitive impairment. Natl Med J China. (2018) 17:1294–301. 10.3760/cma.j.issn.0376-2491.2018.17.003

[11] Winblad B Palmer K Kivipelto M Jelic V Fratiglioni L Wahlund-O. . Mild cognitive impairment–beyond controversies, towards a consensus: report of the International Working Group on Mild Cognitive Impairment. J Intern Med. (2004) 256:240–6. 10.1111/j.1365-2796.2004.01380.x 15324367

[12] Petersen RC Lopez O Armstrong MJ Getchius TSD Mary Ganguli DG Gary S Gronseth DM . Practice guideline update summary: mild cognitive impairment: report of the guideline development, dissemination, and implementation subcommittee of the American Academy of Neurology. Neurology. (2018) 90:126–35. 10.1212/WNL.0000000000004826 29282327 PMC5772157

[13] Kandiah N Christopher YFC. Strategies for the use of Ginkgo biloba extract, EGb 761 ^®^, in the treatment and management of mild cognitive impairment in Asia: expert consensus. CNS Neurosci Ther. (2021) 27:149–62. 10.1111/cns.13536 33352000 PMC7816207

[14] Brouwers MC Florez ID McNair SA Vella ET Yao X. Clinical practice guidelines: tools to support high quality patient care. Semin Nucl Med. (2019) 49:145–52. 10.1053/j.semnuclmed.2018.11.001 30819394

[15] Brouwers MC Kho ME Browman GP Burgers JS Cluzeau F Feder . Development of the AGREE II, part 1: performance, usefulness and areas for improvement. Can Med Assoc J. (2010) 182:1045–52. 10.1503/cmaj.091714 20513780 PMC2900328

[16] Brouwers MC Kho ME Browman GP Burgers JS Cluzeau F Feder G . Development of the AGREE II, part 2: assessment of validity of items and tools to support application. Can Med Assoc J. (2010) 182:E472–8. 10.1503/cmaj.091716 20513779 PMC2900368

[17] Brouwers MC Spithoff K Lavis J Kho ME Makarski J Florez ID. What to do with all the AGREEs? The AGREE portfolio of tools to support the guideline enterprise. J Clin Epidemiol. (2020) 125:191–7. 10.1016/j.jclinepi.2020.05.025 32473992

[18] Kaper NM Heijden GJMG Cuijpers SH Stokroos RJ Aarts MCJ. A comparison of international clinical practice guidelines on adult chronic rhinosinusitis shows considerable variability of recommendations for diagnosis and treatment. Eur Arch Otorhinolaryngol. (2020) 277:659–68. 10.1007/s00405-019-05752-7 31845037

[19] Tian J Xie H Qin B Fan D Shi J Wang L. Chinese diagnostic guidelines of vascular mild cognitive impairment. Chin J Intern Med. (2016) 3:249–56.

[20] WHO. Risk Reduction of Cognitive Decline and Dementia: WHO Guidelines. Geneva: World Health Organization (2019). 31219687

[21] Brien JTO Holmes C Jones M Jones R Livingston G McKeith I . Clinical practice with anti-dementia drugs: a revised (third) consensus statement from the British Association for Psychopharmacology. J Psychopharmacol. (2017) 31:147–68. 10.1177/0269881116680924 28103749

[22] Herukka SK Simonsen AH Andreasen N Baldeiras I Bjerke M Blennow K . Recommendations for cerebrospinal fluid Alzheimer's disease biomarkers in the diagnostic evaluation of mild cognitive impairment. Alzheimers Dement. (2017) 13:285–95. 10.1016/j.jalz.2016.09.009 28341066

[23] Nobili F Arbizu J Bouwman F Drzezga A Agosta F Nestor P . European Association of Nuclear Medicine and European Academy of Neurology recommendations for the use of brain (18) F-fluorodeoxyglucose positron emission tomography in neurodegenerative cognitive impairment and dementia: Delphi consensus. Eur J Neurol. (2018) 25:1201–17. 10.1111/ene.13728 29932266

[24] Cummings J Passmore P McGuinness B Mok V Chen C Engelborghs S . Souvenaid in the management of mild cognitive impairment: an expert consensus opinion. Alzheimer's Res Ther. (2019) 1:73. 10.1186/s13195-019-0528-6 31421681 PMC6698334

[25] Jia J. Writing Group of Chinese Expert Consensus of Cognitive Training, and Chinese Medical Doctor Association Neurologist Branch Cognitive Disorders Professional Committee (2019). Chinese expert consensus of cognitive training. Natl Med J China 99, 4–8. 10.3760/cma.j.issn.0376-2491.2019.01.002

[26] Ismail Z Richard SEB. Recommendations of the 5th Canadian Consensus Conference on the diagnosis and treatment of dementia. Alzheimers Dement. (2020) 16:1182–95. 10.1002/alz.12105 32725777 PMC7984031

[27] Zhou X Huang J Xie M Wu C. Expert consensus of vascular mild cognitive impairment in Chinese traditional medicine. Chin J Inform Trad Chin Med. (2020) 27:1–5. 10.3969/j.issn.1005-5304.201910102

[28] Dunne RA Aarsland D O'Brien JT Ballard C Banerjee S Fox NC . Mild cognitive impairment: the Manchester consensus. Age Ageing. (2021) 50:72–80. 10.1093/ageing/afaa228 33197937 PMC7793599

[29] Breton A Casey D Arnaoutoglou NA. Cognitive tests for the detection of mild cognitive impairment (MCI), the prodromal stage of dementia: meta-analysis of diagnostic accuracy studies. Int J Geriatr Psychiatry. (2019) 34:233–42. 10.1002/gps.5016 30370616

[30] Pinto TCC Machado L Costa MLG Santos MSP Bulgacov TM Rolim APP . Accuracy and psychometric properties of the Brazilian version of the montreal cognitive assessment as a brief screening tool for mild cognitive impairment and alzheimer's disease in the initial stages in the elderly. Dement Geriatr Cogn Disord. (2019) 47:366–74. 10.1159/000501308 31466064

[31] Ciesielska N Sokołowski R Mazur E Podhorecka M Polak-Szabela A Kedziora-Kornatowska K. Is the Montreal Cognitive Assessment (MoCA) test better suited than the Mini-Mental State Examination (MMSE) in mild cognitive impairment (MCI) detection among people aged over 60? Meta-analysis. Psychiatr Pol. (2016) 31:1039–52. 10.12740/PP/45368 27992895

[32] Pinto TCC Machado L Bulgacov TM Rodrigues-Júnior AL Costa MLG Ximenes RCC . Is the Montreal Cognitive Assessment (MoCA) screening superior to the Mini-Mental State Examination (MMSE) in the detection of mild cognitive impairment (MCI) and Alzheimer's Disease (AD) in the elderly? Int Psychogeriatr. (2019) 31:491–504. 10.1017/S1041610218001370 30426911

[33] O'Driscoll C Shaikh M. Cross-cultural applicability of the Montreal cognitive assessment (MoCA): a systematic review. J Alzheimers Dis. (2017) 58:789–801. 10.3233/JAD-161042 28482634

[34] Jack C. R. Jr. Wiste HJ Vemuri P Weigand SD Senjem ML Zeng G . Brain beta-amyloid measures and magnetic resonance imaging atrophy both predict time-to-progression from mild cognitive impairment to Alzheimer's disease. Brain. (2010) 133:3336–48. 10.1093/brain/awq277 20935035 PMC2965425

[35] Diniz BS Pinto Júnior JA Forlenza OV. Do CSF total tau, phosphorylated tau, and beta-amyloid 42 help to predict progression of mild cognitive impairment to Alzheimer's disease? A systematic review and meta-analysis of the literature. World J Biol Psychiatry. (2008) 9:172–82. 10.1080/15622970701535502 17886169

[36] Ewers M Walsh C Trojanowski JQ Shaw LM Petersen RC Jack R Jr. . Prediction of conversion from mild cognitive impairment to Alzheimer's disease dementia based upon biomarkers and neuropsychological test performance. Neurobiol Aging. (2012) 33:1203–14. 10.1016/j.neurobiolaging.2010.10.019 21159408 PMC3328615

[37] Hameed S Fuh JL Senanarong V Ebenezer EGM Looi I Dominguez JC . Role of fluid biomarkers and PET imaging in early diagnosis and its clinical implication in the management of Alzheimer's disease. J Alzheimers Dis Rep. (2020) 4:21–37. 10.3233/ADR-190143 32206755 PMC7081089

[38] Yang G Wang Y Sun J Zhang K Liu J. Ginkgo Biloba for mild cognitive impairment and Alzheimer's disease: a systematic review and meta-analysis of randomized controlled trials. Curr Top Med Chem. (2016) 16:520–8. 10.2174/1568026615666150813143520 26268332

[39] Zhang J Yang C Wei D Li H Leung E. L.-H. . Long-term efficacy of Chinese medicine Bushen Capsule on cognition and brain activity in patients with amnestic mild cognitive impairment. Pharmacol Res. (2019) 146:104319. 10.1016/j.phrs.2019.104319 31220560

[40] Lee J. Effects of aerobic and resistance exercise interventions on cognitive and physiologic adaptations for older adults with mild cognitive impairment: a systematic review and meta-analysis of randomized control trials. Int J Environ Res Public Health. (2020) 17:9216. 10.3390/ijerph,17249216 33317169 PMC7764103

[41] Eyre HA Siddarth P Acevedo B Dyk KV Paholpak P Ercoli L . A randomized controlled trial of Kundalini yoga in mild cognitive impairment. Int Psychogeriatr. (2017) 29:557–67. 10.1017/S1041610216002155 28088925 PMC5540331

[42] Zheng W Xiang YQ Ungvari GS Chiu HFK Ning P Yu X . Tai chi for mild cognitive impairment: a systematic review. Psychogeriatrics. (2017) 17:514–6. 10.1111/psyg.12269 28703397

[43] Tao J Liu J Chen X Xia R Li M Huang M . Mind-body exercise improves cognitive function and modulates the function and structure of the hippocampus and anterior cingulate cortex in patients with mild cognitive impairment. Neuroimage Clin. (2019) 23:101834. 10.1016/j.nicl.2019.101834 31128522 PMC6535682

[44] Yang J Zhang L Tang Q Wang F Li Y Peng H . Tai Chi is effective in delaying cognitive decline in older adults with mild cognitive impairment: evidence from a systematic review and meta-analysis. Evid Based Complement Alternat Med. (2020) 2020:3620534. 10.1155/2020/3620534 32308706 PMC7132349

