# Supplementary material for: Diagnosis and Treatment for Mild Cognitive Impairment: A Systematic Review of Clinical Practice Guidelines and Consensus Statements
Source: Front Neurol. 2021 Oct 12;12:719849. doi: 10.3389/fneur.2021.719849 (PMC8545868; doi:10.3389/fneur.2021.719849)
Supplement: Supplementary file 1 [file Table_1.DOCX]

Table A. Search strategy:

| Pubmed | 1 "Guideline" [Publication Type]  2 guideline [Title/Abstract]  3 expert consensus [Title/Abstract]  4 recommendation statement [Title/Abstract]  5 #1 OR #2 OR #3 OR #4  6 Cognitive Dysfunction [Mesh]  7 cognitive dysfunction [Title/Abstract]  8 mild cognitive impairment*[Title/Abstract]  9 MCI[Title/Abstract]  10 cognitive disorder[Title/Abstract]  11 cognitive impairment[Title/Abstract]  12 neurocognitive disorder[Title/Abstract]  13 cognitive decline[Title/Abstract]  14 mental deterioration*[Title/Abstract]  15 #6 OR #7 OR #8 OR #9 OR #10 OR #11 OR #12 OR #13 OR #14  16 #5 AND #15 |
| --- | --- |
| EMBASE | #1 ‘mild cognitive impairment’/exp  #2 ‘cognitive dysfunction*’:ab,ti OR ‘mci’:ab,ti OR ‘mild cognitive impairment*’:ab,ti OR ‘cognitive disorder’:ab,ti OR ‘cognitive impairment’:ab,ti OR ‘neurocognitive disorder’:ab,ti OR ‘cognitive decline’:ab,ti OR ‘mental deterioration*’:ab,ti  #3 #1 OR #2  #4 ‘guideline’/exp  #5 ‘guideline’:ab,ti OR ‘expert consensus’:ab,ti OR ‘recommendation statement’:ab,ti  #6 #4 OR#5  #7 #3 AND #6 |
| China National Knowledge Infrastructure | （认知障碍 + 认知功能障碍 + 轻度认知障碍 + 轻度认知功能障碍 + 轻度神经认知障碍 + 认知减退 + 认知损害 + 轻度认知损害 + 轻度认知损伤 + 轻微认知功能损害 + 轻度认知功能损害 + 精神衰退）AND （指南 + 共识 + 推荐 + 建议） |
| Wanfang Database | ("认知功能障碍"+"轻度认知障碍"+"轻度认知功能障碍"+"轻度神经认知障碍"+"认知减退"+"认知损害"+"轻度认知损害"+"轻度认知损伤"+"轻微认知功能损害"+"轻度认知功能损害"+"精神衰退") AND ("指南"+"共识"+"推荐"+"建议") |
| Chinese Science and Technology Periodical Database | ("认知功能障碍"+"轻度认知障碍"+"轻度认知功能障碍"+"轻度神经认知障碍"+"认知减退"+"认知损害"+"轻度认知损害"+"轻度认知损伤"+"轻微认知功能损害"+"轻度认知功能损害"+"精神衰退") AND ("指南"+"共识"+"推荐"+"建议") |
| Chinese Biological Medicine Database | ( "认知功能障碍"[常用字段:智能] OR "轻度认知障碍"[常用字段:智能] OR "轻度认知功能障碍"[常用字段:智能] OR "轻度神经认知障碍"[常用字段:智能] OR "认知减退"[常用字段:智能] OR "认知损害"[常用字段:智能] OR "轻度认知损害"[常用字段:智能] OR "轻度认知损伤"[常用字段:智能] OR "轻微认知功能损害"[常用字段:智能] OR "轻度认知功能损害"[常用字段:智能] OR "精神衰退"[常用字段:智能]) AND （"指南"[常用字段:智能] OR "共识"[常用字段:智能] OR "推荐 "[常用字段:智能] OR "建议"[常用字段:智能]） |
