# Supplementary material for: Diagnosis and Treatment for Mild Cognitive Impairment: A Systematic Review of Clinical Practice Guidelines and Consensus Statements
Source: Front Neurol. 2021 Oct 12;12:719849. doi: 10.3389/fneur.2021.719849 (PMC8545868; doi:10.3389/fneur.2021.719849)
Supplement: Supplementary file 2 [file Table_2.DOCX]

**TABLE B** Summary of grading systems used in included guidelines and consensus statement

| Guidelines | Level of evidence | Level of recommendation |
| --- | --- | --- |
| Tian *et al.* (Tian et al., 2016) | Diagnostic evidence  I: A prospective study of a broad spectrum of persons at risk for developing the outcome. (The outcome is defined by an acceptable reference standard for case definition. The outcome is objective or measured by an observer who is masked to the presence of the risk factor. Study results allow calculation of measures of prognostic accuracy.)  II: A prospective study of a narrow spectrum of persons at risk for developing the outcome or a retrospective study with large sample. (The outcome is defined by an acceptable reference standard for case definition. The outcome is objective or measured by an observer who is masked to the presence of the risk factor. Study results allow calculation of measures of prognostic accuracy.)  III：A retrospective study with small sample of the case or control subjects (The outcome is objective or measured by an observer who is masked to the presence of the risk factor.)  IV: Any study of which outcome is not objective or measured by an observer who is masked to the presence of the risk factor, expert opinion, or descriptive case study without control.  诊断性证据： I级：在广泛可疑人群进行的前瞻性研究（要求使用"金标准"定义病例，采用了盲法评估，能够进行诊断精确性评估） II级：在非广泛可疑人群进行的前瞻性研究，或样本量较大的回顾性研究（要求使用"金标准"定义病例，采用了盲法评估，能够进行诊断精确性评估） III级：回顾性研究（病例组或是对照组样本不大，要求采用盲法评估） IV级：非盲法评估的任何设计，或专家观点，或无对照的描述性病例系列 Therapeutic evidence  I: A high-quality prospective randomized controlled clinical trial in a representative population or A high-quality systematic review based on prospective randomized controlled clinical trial in a representative population.  The following are also required:  a. randomized control and concealed allocation  b. with primary outcomes specified  c. exclusion/inclusion criteria clearly defined  d. adequate accounting for dropouts and crossovers with numbers sufficiently low to have minimal potential for bias.  e. detailed records of baseline characteristics with comparability between groups or differences with statistical correction  II a prospective matched cohort study with masked or objective outcome assessment in a representative population that meets b−e above (see Class I) or an RCT of the intervention of interest in a representative population that lacks one criteria a–e above (see Class I).  III: All other controlled trials (including well-defined natural history controls and before-after studies) in a representative population. Outcome assessment is masked, objective, or performed by someone who is not a member of the treatment team.  IV: Uncontrolled studies, case series, case report, and expert advice.  干预性证据水平： I类证据：典型人群进行的高质量前瞻性随机对照盲法试验，或对典型人群进行的前瞻性随机对照盲法试验的高质量系统综述。须符合下述标准： 1.随机隐藏 2.有明确定义的主要结局 3.有明确定义的纳入/排除标准 4.详细记录脱落病例，交叉设计的脱落病例不至于造成明细偏倚 5.详细记录了基线特征且组间具有可比性，或存在差异但进行了统计学校正 II类证据：典型人群进行的前瞻性组间匹配的队列研究，结局评估采用了盲法，符合上述2~5项的标准；或典型人群的随机对照试验，但上述1-5项标准有一项不符合， III类证据：典型病例中进行的所有其他对照试验（包括良好的自然病程对照或患者自身对照）证实，结局评估与患者治疗应该独立 IV类证据：非对照研究、病例系列、病例报告或专家观点 | Diagnostic level of recommendation  A: (definitely helpful/predictable, unhelpful/unpredictable) with one study or more studies of level Ⅰ，or two studies of level Ⅱ with consistent results  B: (most likely to be helpful/predictable, unhelpful/unpredictable) with at least one study of level II，or multiple studies of level III.  C: (most likely to be helpful/predictable, unhelpful/unpredictable) with at least two study of level III.  D: Lacking good clinical studies, or studies of level IV  诊断性推荐强度： A级：（肯定有帮助/可预测、无帮助/不可预测）至少一个Ⅰ类研究或两个结果一致的Ⅱ类研究 B级：（很可能有帮助/可预测、无帮助/不可预测）至少一个II类研究或大量的Ⅲ类研究 C级：（很可能有帮助/可预测、无帮助/不可预测）至少两个III.类研究 D级：缺乏好的临床研究证据，或Ⅳ类证据 Therapeutic level of recommendation  A: (surely effective, ineffective or harmful) with one study or more studies of level Ⅰ，or at least two studies of level Ⅱ with consistent results  B: (likely to be effective, ineffective or harmful) with at least one study of level II，or multiple studies of level III.  C: (may be effective, ineffective or harmful) with at least two study of level III.  D: Lacking good clinical studies, or studies of level IV  干预性推荐强度： A级推荐（肯定有效、无效或有害）至少一个Ⅰ类研究，或至少两个结果一致的Ⅱ类研究 B级推荐（很可能有效、无效或有害）至少一个Ⅱ类研究，或大量的Ⅲ类研究 C级推荐（可能有效、无效或有害）至少两个Ⅲ类研究 D级推荐缺乏好的临床研究证据或Ⅳ类研究 |
| Petersen *et al.*(Petersen et al., 2018) (AAN) | Screening scheme  Class I  A statistical, population-based sample of patients studied at a uniform point in time (usually early) during the course of the condition. All patients undergo the intervention of interest. The outcome, if not objective, is determined in an evaluation that is masked to the patients’ clinical presentations.  Class II  A statistical, non-referral-clinic-based sample of patients studied at a uniform point in time (usually early) during the course of the condition. Most patients undergo the intervention of interest. The outcome, if not objective, is determined in an evaluation that is masked to the patients’ clinical presentations.  Class III  A sample of patients studied during the course of the condition. Some patients undergo the intervention of interest. The outcome, if not objective, is determined in an evaluation by someone other than the treating physician.  Class IV  Studies not meeting Class I, II, or III criteria, including consensus, expert opinion, or a case report.  Prognostic accuracy scheme  Class I A cohort study of a broad spectrum of persons at risk for developing the outcome (e.g., target disease, work status). The outcome is defined by an acceptable reference standard for case definition. The outcome is objective or measured by an observer who is masked to the presence of the risk factor. Study results allow calculation of measures of prognostic accuracy.  Class II  A case-control study of a broad spectrum of persons with the condition compared with a broad spectrum of controls, or a cohort study of a broad spectrum of persons at risk for the outcome (e.g., target disease, work status) where the data were collected retrospectively. The outcome is defined by an acceptable reference standard for case definition. The outcome is objective or measured by an observer who is masked to the presence of the risk factor. Study results allow calculation of measures of prognostic accuracy.  Class III  A case-control study or a cohort study where either the persons with the condition or the controls are of a narrow spectrum where the data were collected retrospectively. The outcome is defined by an acceptable reference standard for case definition. The outcome is objective or measured by an observer who did not determine the presence of the risk factor. Study results allow calculation of measures of a prognostic accuracy.  Class IV  Studies not meeting Class I, II, or III criteria, including consensus, expert opinion, or a case report.  Therapeutic scheme  Class I  A randomized controlled clinical trial of the intervention of interest with masked or objective outcome assessment, in a representative population. Relevant baseline characteristics are presented and substantially equivalent between treatment groups, or there is appropriate statistical adjustment for differences.  The following are also required:  a. concealed allocation  b. no more than 2 primary outcomes specified  c. exclusion/inclusion criteria clearly defined  d. adequate accounting for dropouts (with at least 80% of enrolled subjects completing the study) and crossovers with numbers sufficiently low to have minimal potential for bias.  e. For noninferiority or equivalence trials claiming to prove efficacy for one or both drugs, the following are also required*:  i. The authors explicitly state the clinically meaningful difference to be excluded by defining the threshold for equivalence or noninferiority.  ii. The standard treatment used in the study is substantially similar to that used in previous studies establishing efficacy of the standard treatment (e.g., for a drug, the mode of administration, dose, and dosage adjustments are similar to those previously shown to be effective).  iii. The inclusion and exclusion criteria for patient selection and the outcomes of patients on the standard treatment are comparable to those of previous studies establishing efficacy of the standard treatment.  iv. The interpretation of the study results is based upon a per-protocol analysis that accounts for dropouts or crossovers.  f. For crossover trials, both period and carryover effects examined and statistical adjustments performed, if appropriate  Class II  An RCT of the intervention of interest in a representative population with masked or objective outcome assessment that lacks one criteria a–e above (see Class I) or a prospective matched cohort study with masked or objective outcome assessment in a representative population that meets b−e above (see Class I). (Alternatively, a randomized crossover trial missing 1 of the following 2 characteristics: period and carryover effects described or baseline characteristics of treatment order groups presented.) All relevant baseline characteristics are presented and substantially equivalent among treatment groups, or there is appropriate statistical adjustment for differences.  Class III  All other controlled trials (including studies with external controls such as well-defined natural history controls). (Alternatively, a crossover trial missing both of the following 2 criteria: period and carryover effects described or baseline characteristics of treatment order groups presented.) A description of major confounding differences between treatment groups that could affect outcome.** Outcome assessment is masked, objective, or performed by someone who is not a member of the treatment team.  Class IV  Studies that (1) did not include patients with the disease, (2) did not include patients receiving different interventions, (3) had undefined or unaccepted interventions or outcomes measures, or (4) had no measures of effectiveness or statistical precision presented or calculable.  * Note that numbers 1–3 in Class Ie are required for Class II in equivalence trials. If any 1 of the 3 is missing, the class is automatically downgraded to Class III.  **Objective outcome measurement: an outcome measure that is unlikely to be affected by an observer’s (patient, treating physician, investigator) expectation or bias (e.g., blood tests, administrative outcome data). | A  Level A is the strongest recommendation level and is denoted by the use of the helping verb must. Must recommendations are rare, as they are based on the high confidence in the evidence and require both a high magnitude of benefit and low risk.  B  Level B corresponds to the helping verb should. Should recommendations tend to be more common, as the requirements are less stringent but still based on the  evidence and benefit-risk profile.  C  Level C corresponds to the helping verb may. May recommendations represent the lowest allowable recommendation level the AAN considers useful and accommodate the highest degree of practice variation. |
| Jia *et al.* (Jia et al., 2018) | I. A prospective randomized controlled clinical trial of the intervention of interest with masked or objective outcome assessment, in a representative population or systematic reviews based on the trials above.  The following are also required:  a. randomized control and concealed allocation  b. primary objective outcome clearly defined  c. exclusion/inclusion criteria clearly defined  d. adequate accounting for dropouts and crossovers with numbers sufficiently low to have minimal potential for bias.  e. clearly describing the characteristics of the population in the baseline period which are matched in the treatment group and the control group.  II. A prospective matched cohort study with masked or objective outcome assessment in a representative population that meets b−e above (see Class I) or an RCT of the intervention of interest in a representative population with masked or objective outcome assessment that lacks one criteria a–e above (see Class I)  III. All other controlled trials (including well-defined natural history controls or before-after study) in a representative population.  IV. Uncontrolled studies, case analysis, case report, and expert advice.  I级：有力的前瞻性、随机、对照的临床研究，人群具有代表性，结果评价明确可靠；或者基于以上资料的系统性综述。具体满足以下条件：(a)随机、盲法；(b)主要观察指标明确；(C) 纳入标准和排除标准明确；(d)脱失率低，不会造成结果的偏倚；(e)明确描述人群基线期的特征，而且基线期的特征在治疗组和对照组匹配。  II级：前瞻性的、匹配的队列研究，人群具有代表性，结果评价明确可靠，符合以上I级证据中 a—e点；或者一个随机、对照研究，人群具有代表性，但不符合 a～e中的某一条件。  III级：其他对照研究 (包括描述明确的自然病程对照研究或自身对照研究)，人群具有代表性。  IV级：非对照研究，病例分析，个案报道，专家意见。 | A. with one study or more convictive studies of level Ⅰ，or two studies or more convictive studies level Ⅱ with consistent results  B. with one study or more convictive studies of level Ⅱ，or multiple studies of level Ⅲ  C. with two studies or more convictive studies level Ⅲ  Expert consensus: study's conclusion related to clinical treatment or consistent expert recommendation  A级(结果确定 )：至少1个有说服力的I级证据，或者至少 2个结论一致的、有说服力的Ⅱ级证据。  B级 (结果很可能 )：至少1个有说服力的Ⅱ级证据 ，或大量的Ⅲ级证据 。  C级(结果可能 )：至少2个有说服力的Ⅲ级证据。  专家共识：不符合上述推荐标准，但与临床治疗紧密相关 的研究结论，或专家的一致意见。 |
| Jia JP *et al.* (Jia et al., 2019) | N/A | N/A |
| Zhou *et al.* (Zhou et al., 2020) | Ⅰ. Large sample size and randomized controlled trial with clear result and low errors of false positive and false negative.  Ⅱ. Small sample size and randomized controlled trial with unclear result and high errors of false positive and false negative.  Ⅲ. Non-randomized controlled trial, prospective control trial, and expert consensus.  Ⅳ. Non-randomized controlled trial, retrospective control trial, and expert consensus.  Ⅴ. Case report, uncontrolled studies and expert recommendation.  Ⅰ.大样本，随机研究，结果清晰，假阳性或假阴性的错误很低  Ⅱ.小样本，随机研究，结果不确定，假阳性和/或假阴性的错误较高  Ⅲ.非随机，同期对照研究和古今中医专家共识  Ⅳ.非随机，历史对照和当代中医专家共识  Ⅴ.病例报道，非对照研究和专家意见 | A. with two studies or more studies of level Ⅰ  B. with one study of level Ⅰ  C. with studies of level Ⅱ  D. with one study or more studies of level Ⅲ  E. with studies of level Ⅳ and Ⅴ  A.至少有 2 项Ⅰ级研究结果支持  B.仅有 1 项 Ⅰ 级研究结果支持  C.仅有Ⅱ级研究结果支持  D.至少有 1 项Ⅲ级研究结果支持  E.仅有Ⅳ级或Ⅴ级研究结果支持 |
| O’Brien *et al.(Brien et al., 2017)* | Categories of evidence for causal relationships and treatment  I Evidence from meta-analysis of randomised controlled trials,^b^ at least one large, good quality, randomised controlled trial^b^ or replicated, smaller, randomized controlled trials^b^  II Evidence from small, non-replicated, randomised controlled trials,^b^ at least one controlled study without randomisation or evidence from at least one other type of quasi-experimental study  III Evidence from non-experimental descriptive studies, such as uncontrolled, comparative, correlation and case-control studies IV Evidence from expert committee reports or opinions and/or clinical experience of respected authorities  Proposed categories of evidence for non-causal relationships  I Evidence from large representative population samples  II Evidence from small, well designed, but not necessarily representative samples  III Evidence from non-representative surveys, case reports  IV Evidence from expert committee reports or opinions and/or clinical experience of respected authorities  ^b^Randomised controlled trials must have an appropriate control treatment arm; for primary efficacy this should include a placebo condition.  ^c^ Extrapolation may be necessary because of evidence that is only indirectly related, covers only a part or the area of practice under consideration, has methodological problems or is contradictory. | A Directly based on category I evidence  B Directly based on category II evidence or extrapolated^c^ recommendation from category I evidence  C Directly based on category III evidence or extrapolated^c^ recommendation from category I or II evidence  D Directly based on category IV evidence or extrapolated^c^ recommendation from category I, II or III evidence |
| Cummings *et al.(Cummings et al., 2019)* | N/A | N/A |
| WHO(WHO, 2019) | Grading of Recommendations Assessment, Development and Evaluation (GRADE) | Strong recommendations imply that most individuals would want the intervention and should receive it.  Conditional recommendations imply that different choices may be appropriate for individual patients and they may require assistance at arriving at management decisions. |
| Ismail *et al.(Ismail and Richard, 2020)* | Quality of evidence  A High: “further research is unlikely to change confidence in the estimate of effect”  B Moderate: “further research is likely to have an important impact on the confidence in the estimate of effect and may change the estimate”  C Low: “further research is very likely to have an important impact on the confidence in the estimate of effect and is likely to change the estimate” | Strength of recommendation  1 Strong: benefits clearly outweigh undesirable effects  2 Weak, or conditional: either lower quality evidence or desirable and undesirable effects are more closely balanced |
| Dunne *et al.(Dunne et al., 2021)* | N/A | N/A |
| Kandiah *et al.(Kandiah and Christopher, 2021)* | A: Data derived from multiple randomized, placebo-controlled clinical trials, or meta-analyses  B: Data derived from a single randomized clinical trial or large nonrandomized studies  C: Consensus of opinion of experts and/or case reports, small studies, retrospective studies | Class I: Evidence and/or general agreement that a given treatment or procedure is beneficial, useful, effective (is recommended/is indicated)  Class IIa: Weight of evidence/opinion is in favor of usefulness/efficacy (is reasonable to consider)  Class IIb: Usefulness/efficacy is less well established by evidence/opinion (may be reasonable to consider)  Class III: Evidence or general agreement that the given treatment or procedure is not useful/effective, or in some cases may be harmful (is not recommended) |
| Herukka et al (Herukka et al., 2017) | The quality of the evidence for each relevant outcome was graded as “high”, “moderate”, “low”, or “very low” | N/A |
| Nobili et al (Nobili et al., 2018) | Based on the resulting assessment, the quality of evidence was then ranked within the 21 PICOs. More precisely, PICOs lacking critical outcomes entirely were put at the lowest level, while those with soundest methodology, numerous studies, large total number of included subjects, and large and consistent effect size and were graded best. The other PICOs were ranked in between. In this way, we provided information about relative availability of evidence, classified in four levels as “very poor/lacking”, “poor”, “fair” and “good” | N/A |

N/A: not applicable

**Reference**

Brien, J.T.O., Jones, C.H.M., Livingston, G., Mittler, I.M.P., Ritchie, C., L, L.R.E., et al. (2017). Clinical practice with anti-dementia drugs: A revised (third) consensus statement from the British Association for Psychopharmacology. *Journal of Psychopharmacology* 31(2)**,** 147-168.

Cummings, J., Passmore, P., McGuinness, B., Mok, V., Chen, C., Engelborghs, S., et al. (2019). Souvenaid in the management of mild cognitive impairment: An expert consensus opinion. *Alzheimer's Research and Therapy* 1(11)**,** 73.

Dunne, R.A., Aarsland, D., O'Brien, J.T., Ballard, C., Banerjee, S., Fox, N.C., et al. (2021). Mild cognitive impairment: the Manchester consensus. *AGE AND AGEING* 50(1)**,** 72-80.

Herukka, S.K., Simonsen, A.H., Andreasen, N., Baldeiras, I., Bjerke, M., Blennow, K., et al. (2017). Recommendations for cerebrospinal fluid Alzheimer's disease biomarkers in the diagnostic evaluation of mild cognitive impairment. *Alzheimers Dement* 13(3)**,** 285-295. doi: 10.1016/j.jalz.2016.09.009.

Ismail, Z., and Richard, S.E.B. (2020). Recommendations of the 5th Canadian Consensus Conference on the diagnosis and treatment of dementia. *Alzheimers Dement* 16(8)**,** 1182-1195.

Jia, J., Writing Group of Chinese Expert Consensus of Cognitive Training, and Chinese Medical Doctor Association Neurologist Branch Cognitive Disorders Professional Committee (2019). Chinese expert consensus of cognitive training. *Natl Med J China* 99(1)**,** 4-8.

Jia, J., Writing group of Chinese guidelines for diagnosis and treatment of dementia and cognitive impairment, and Chinese Medical Doctor Association Neurologist Branch Cognitive Disorders Professional Committee (2018). Chinese guidelines for diagnosis and treatment of dementia and cognitive impairment in 2018 (five): diagnosis and treatment of mild cognitive impairment. *Natl Med J China* 17**,** 1294-1301.

Kandiah, N., and Christopher, Y.F.C. (2021). Strategies for the use of Ginkgo biloba extract, EGb 761 ® , in the treatment and management of mild cognitive impairment in Asia: Expert consensus. *CNS NEUROSCI THER* 27(2)**,** 149-162.

Nobili, F., Arbizu, J., Bouwman, F., Drzezga, A., Agosta, F., Nestor, P., et al. (2018). European Association of Nuclear Medicine and European Academy of Neurology recommendations for the use of brain (18) F-fluorodeoxyglucose positron emission tomography in neurodegenerative cognitive impairment and dementia: Delphi consensus. *Eur J Neurol* 25(10)**,** 1201-1217. doi: 10.1111/ene.13728.

Petersen, R.C., Lopez, O., Armstrong, M.J., Getchius, T.S.D., Mary Ganguli, D.G., Gary S Gronseth, D.M., et al. (2018). Practice guideline update summary: Mild cognitive impairment: Report of the Guideline Development, Dissemination, and Implementation Subcommittee of the American Academy of Neurology. *Neurology* 90(3)**,** 126-135. doi: 10.1212/WNL.0000000000004826.

Tian, J., xie, H., Qin, B., Fan, D., Shi, J., and Wang, L. (2016). Chinese diagnostic guidelines of vascular mild cognitive impairment. *Chin J Intern Med* 3**,** 249-256.

WHO (2019). "Risk reduction of cognitive decline and dementia: WHO guidelines". (Geneva: World Health Organization).

Zhou, X., Huang, J., Xie, M., and Wu, C. (2020). Expert consensus of vascular mild cognitive impairment in Chinese traditional medicine. *Chinese Journal of Information on Traditional Chinese Medicine* 27(3)**,** 1-5.
