# Supplementary material for: Diagnosis and Treatment for Mild Cognitive Impairment: A Systematic Review of Clinical Practice Guidelines and Consensus Statements
Source: Front Neurol. 2021 Oct 12;12:719849. doi: 10.3389/fneur.2021.719849 (PMC8545868; doi:10.3389/fneur.2021.719849)
Supplement: Supplementary file 3 [file Table_3.DOC]

**TABLE C1** Recommendations for screening and diagnosis

| Recommendations |  |
| --- | --- |
| **Diagnostic criteria**  —Use VAS-COG Society criteria, DSM5, Vascular Impairment of Cognition Classification Consensus Study or the American Heart Association consensus statement for diagnosis of VaMCI (*Ismail et al*); VAS-COG criteria refine the type and severity of the evidence on cerebrovascular disease, supplements the supporting characteristics of people without a history of cerebrovascular events, and is more operable than the AHA/ASA standard (*Tian et al*).  —The diagnosis of MCI should include subtype (*Jia et al*). | |
| **Identifying Risk factors**  —Clinicians should perform medical evaluation for MCI risk factors that are potentially modifiable (*Dunne et al; Petersen et al*); the potentially modifiable risk factors to cognitive dysfunction include but not limited to the treatment of physical illness, depression and other psychiatric disorders, isolation, optimization of hearing and visual disturbance, recommendations and interventions to promote alcohol cessation, and rationalization of medications (e.g. anticholinergics, hypnotics and opiates) (*Dunne et al*). | |
| **Clinical history**  —Clinical history can’t rely solely on the self-report from patients and should be supplemented by familiar people (*Tian et al; Jia et al*).  —Clinical history should be combined with neuropsychological testing when making diagnosis (*Petersen et al; Cummings et al; Ismail et al*); physicians should explore the clinical history because it provides important information about the changes in individual patients, which may alert them to the emerging cognitive impairment even when an objective screening test is normal (*Cummings et al*); clinicians should use validated assessment tools (*Petersen et al*). | |
| **Neuropsychological testing**  —Clinical history should be combined with neuropsychological testing when making diagnosis (*Petersen et al; Cummings et al; Ismail et al*);  —Cognitive assessment should include one comprehensive cognitive test and at least four single domain tests, such as executive, memory, language, and visual spatial function should be performed (*Tian et al*); all MCI patients should be tested for overall cognitive function or multiple cognitive domains, and targeted selection can be made based on clinical prompts (*Jia et al*);  —Patients with MCI should have equitable access to neuropsychological testing and expertise (*Dunne et al*).  —Cognitive testing to screen asymptomatic adults for the presence of mild cognitive impairment or dementia, including asymptomatic persons with risk factors such as family history or vascular risk factors, is not recommended (*Ismail et al*).  —**Cognitive testing:**   - If more time is allowed, preference should be given to using a more comprehensive psychometric screening tool (the Modified Mini-Mental State [3MS] examination, the Mini-Mental State Examination [MMSE], or the Rowland Universal dementia assessment scale [RUDAS]) (*Ismail et al*). - MMSE remains the most widely used instrument, with high sensitivity and specificity for separating moderate dementia from normal cognition and is recommended in many countries. However, it lacks sensitivity for the diagnosis of mild dementia or MCI (*Ismail et al*); application of MMSE alone is not sensitive to MCI and can be combined with other inspections to increase sensitivity (*Jia et al*). - The MoCA is more sensitive to MCI than the MMSE and its use is recommended when mild cognitive impairment is suspected or in cases where there is suspicion of cognitive impairment or concern about the patient’s cognitive status, and the MMSE score is in the “normal” range (24+ out of 30) (*Ismail et al*); cognitive screening tools exist specifically for the early identification of MCI (MoCA, TorCA). Among them, the MoCA offers strong normative data while the TorCA has just been recently published (*Ismail et al*); MoCA has high sensitivity and specificity in identifying MCI and can be used for early screening of MCI (*Jia et al*). - FCSRT has good sensitivity and specificity in the identification of MCI, and is suitable for the diagnosis and identification of MCI (*Jia et al*). - NINDS-CSN has three neuropsychological assessment schemes intended for different needs, of which the 5-minute scheme including memory, orientation, and language assessment is simple and easy to use, and can be used for rapid VaMCI screening, but should be supplemented with tests of executive function and overall cognition (*Tian et al*).   —**Activities of daily living testing and functional assessment:**   - It is recommended that clinicians assess for MCI using validated tools, including testing for functional assessment (ADL) (*Kandiah et al*). - All patients with MCI should be tested for instrumental daily ability or functional activities questionnaire (FAQ) (*Jia et al*). - LADL ability assessment is indispensable for VaMCI or MCI screening, and should be used as a necessary supplement to clinical screening (*Tian et al*).   —**Behavioral and psychological assessment:** If clinical symptoms suggest mental behavioral symptoms, MCI patients should be evaluated for mental behavioral symptoms to guide diagnosis and treatment (*Jia et al*). | |
| **Use of physical examination for diagnosis and predicting the progression to dementia**  —Physical examination should be carried out (*Tian et al; Jia et al*);  —Some physical examinations, such as gait, smell and hearing, combined with neuropsychological tests can be used to identify the cause of MCI, clarify the accompanying diseases, assist in the early identification of MCI and predict the progress of MCI (*Jia et al*);  —**Dual-task gait**: Some physical examinations, such as gait (*Jia et al*); Dual-task gait impairment (lower speed or high cost) is associated with future incident dementia. In MCI samples, dual-task gait was shown to predict time to progression to dementia. Variability in the delivery of testing protocols is noted. We recommend that dual-task gait test may be used in specialized clinics (memory clinics) to help identify mild cognitive impairment (MCI) older adults at higher risk of progression to dementia if time/resources are available (*Ismail et al*). | |
| **Use of Neuroimaging for diagnosis**  —The imaging evaluation of VaMCI cerebrovascular disease evidence involves cerebral infarction, white matter hyperintensity, hemorrhage, brain atrophy and other five aspects; multiple lacunar cerebral infarction and extensive fusion of white matter lesions are the most common (*Tian et al*);  —**Structural imaging**: Structural imaging is a routine examination technique to confirm VaMCI cerebrovascular evidence (*Tian et al*);  —**Functional imaging-MRI**: MRI of head should be carried out for first-visit MCI patients (*Jia et al*); MRI is more sensitive than CT in detecting cerebral infarction, white matter hyperintensity, microhemorrhage, and brain atrophy (*Tian et al*); MRI's scale for Medial Temporal lobe Atrophy (MTA) can be used to assess hippocampal atrophy (*Tian et al*);  —**Functional imaging-FDG-PET**: FDG-PET can be used to support the diagnosis of AD, FTLD and DLB in MCI; prognosis of AD dementia conversion compared to biomarkers of amyloidosis and may also identify non-Alzheimer types of neurodegeneration early in the course of the disease; the use of FDG-PET to ascertain FTLD in many specialized memory clinics and in dedicated neurological and psychiatric units is already part of clinical routine work-up; ‘FTD phenocopies’ have been identified, i.e. patients with behavioural disturbances of varied aetiologies that mimic FTD but normal metabolism at FDG-PET, and thus a normal FDG-PET is particularly valuable to exclude a neurodegenerative disease; besides FDG-PET’s NPV, the presence of the posterior cingulate island sign (i.e. relatively preserved metabolism in the posterior cingulate area) and occipital hypometabolism at the stage of MCI support a diagnosis of DLB (*Nobili et al*); for patients with MCI whose cause cannot be determined by clinical and structural imaging examinations, if permit, PET examination can be considered to confirm the diagnosis of the cause (*Jia et al*); FDG-PET or PIB-PET can be used to assess glucose metabolism or Aβ deposition to support the clinical diagnosis of AD, and should be used as a differential test for VaMCI (*Tian et al*). | |
| **Use of blood tests for excluding other diseases**  —Hematology tests are recommended for all patients who visit for the first time, such as complete blood count, erythrocyte sedimentation rate, blood electrolytes, blood sugar, liver and kidney function, and thyroxine levels. If necessary, other tests such as vitamin B12, syphilis serology test, HIV and so on can be performed. (*Jia et al*);  —At the time of diagnosis, the blood should be checked for vitamin B12, folic acid, thyroid-stimulating hormone, homocysteine, etc., and serological tests such as Borrelia and HIV should be performed to rule out related diseases (*Tian et al*). | |
| **Use of Biomarker assessments for helping confirm diagnosis**  —Biomarkers may be used to diagnose prodromal AD in patients with MCI (*Cummings et al*); biomarker assessments, including imaging, may help confirm a diagnosis of MCI, and establish the presence of CVD (vascular pathology) (*Kandiah et al*);  —Clinicians should attempt to provide patients with an explanation for their decline in cognition, which in some cases will include using biomarkers for the early detection of neurodegeneration (*Dunne et al*);  —Given the heterogeneity of MCI, decisions about whom to investigate and the depth of that investigation, including the utility of molecular biomarkers, should be made on an individual basis (*Dunne et al*);  —For patients and families asking about biomarkers in MCI, clinicians should counsel that there are no accepted biomarkers available at this time (*Petersen et al*);  —**CSF biomarkers**: CSF biomarkers is recommended to be used in MCI patients as an add-on to clinical evaluation alone for predicting functional decline or progression to AD dementia (*Herukka et al*); for patients with amnestic MCI, the cerebrospinal fluid Tau protein and AB42 can be checked for early detection of AD patients (*Jia et al*); CSF biomarkers could not be recommended as an alternative to FDG-PET or amyloid-PET in predicting future decline or conversion; CSF biomarkers are recommended to be used to inform future disease management, but the strength of this recommendation was weak because of the small amount of evidence (*Herukka et al*); given the complexity of diagnosing patients with MCI and emerging evidence that CSF can aid in prognostication, CSF sampling may be useful on an individual by individual basis in patients with MCI in whom a diagnosis of Alzheimer’s disease is suspected (*Dunne et al*). | |
| **Follow-up of MCI patients or monitoring the change in cognitive status**  —For patients diagnosed with MCI, clinicians should perform serial assessments over time to monitor for changes in cognitive status (*Petersen et al*) and perform follow-up assessments (*Jia et al*);  —NICE guidelines for the investigation and follow-up of the MCI syndrome are urgently required (*Dunne et al*). | |

Notes: Opposite Recommendations are addressed in red color. Abbreviation: VAS-COG: Vascular Behavioral and Cognitive Disorders; DSM5: Diagnostic and Statistical Manual of Mental Disorders; VaMCI: Vascular mild cognitive impairment; MCI: mild cognitive impairment; 3MS:Modified Mini-Mental State; MMSE: the Mini-Mental State Examination; RUDAS: the Rowland Universal dementia assessment scale; MoCA: Montreal cognitive assessment; TorCA: Toronto Cognitive Assessment; NINDS-CSN: National Institute for Neurological Disorders and Stroke and Canadian Stroke Network; FCSRT: free and cured selective reminding test; MRI: Magnetic resonance imaging; PET: positron emission tomography; CSF: Cerebrospinal fluid

Notes: +: recommended for screening for MCI; –: not recommended for screening in MCI; N/A: not applicable; Green: strong recommendation; Yellow: moderate recommendation; Red: week recommendation; N/A: not applicable

**TABLE C2** Recommendations for treatment and management

| Recommendations |  |
| --- | --- |
| **Interventions for risk reduction** | |
| —**Risk of Alcohol use disorder**: Interventions aimed at reducing or ceasing hazardous and harmful drinking should be offered to adults with normal cognition and mild cognitive impairment to reduce the risk of cognitive decline and/or dementia in addition to other health benefits (*WHO*).  —**Risk of ceasing medications**: For patients diagnosed with MCI, clinicians should wean patients from medications that can contribute to cognitive impairment (where feasible and medically appropriate) and treat modifiable risk factors that may be contributing (*Petersen et al*). | |
| **Pharmacologic interventions**  —**No accepted drugs**: Pharmacologic therapy, except for the treatment of depression or other neuropsychiatric symptoms, is usually not appropriate for patients diagnosed with MCI (*Cummings et al*). Clinicians should counsel the patients and families that there are no pharmacologic or dietary agents currently shown to have symptomatic cognitive benefit in MCI and that no medications are FDA-approved for this purpose (*Petersen et al*); the efficacy of drugs for the treatment of MCI needs to be further confirmed (*Jia et al*).  —**Cholinesterase inhibitors**: Cholinesterase inhibitors are not effective in those with mild cognitive impairment (*Brien et al*), and should be deprescribed (*Ismail et all*); for patients diagnosed with MCI, clinicians may choose not to offer cholinesterase inhibitors (*Petersen et al*). If clinicians choose to offer cholinesterase inhibitors, they must first discuss with patients the fact that this is an off-label prescription not currently backed by empirical evidence (*Petersen et al*). There is a lack of robust evidence supporting the use of AChEI in improving MCI symptoms (*Kandiah et al*).  —**Memantine**: Memantine should be deprescribed for individuals with mild cognitive impairment (*Ismail et all*).  —**EHb761®** : EGb 761® has demonstrated improvement in MCI symptoms, and is the only pharmacological agent recommended in existing guidelines for the symptomatic treatment of MCI. It is clinically appropriate to incorporate EGb 761® as part of the multidomain intervention for MCI. EGb 761® may improve cognitive performance in MCI patients. Due to its beneficial effects on cerebrovascular blood flow, it is reasonable to expect that EGb 761® may benefit MCI patients with CVD. EGb 761® may help delay progression of MCI to dementia in some individuals (*Kandiah et al*).  —**Chinese herbal decoction**: Different Chinese herbal decoction are advised based on syndrome differentiation; for deficiency of qi and blood syndrome: Guipi decoction; for deficiency of kidney essence syndrome: Heche Dazao pills; for deficiency of kidney and spleen: Huanshaodan; for syndrome of confused by phlegm: Ditan decoction; for syndrome of internal blockade of static blood: Tongqiao Huoxue decoction (*Zhou et al*). | |
| **Non-pharmacologic interventions**  **—Multidomain intervention strategy**: A multidomain intervention strategy is useful in MCI to benefit both neurodegenerative and vascular pathologies (*Kandiah et al*), and is recommended (*Cummings et al*). The strategy should at least include physical exercise, smoking cessation, management of hypertension and diabetes, cognitive training, and psychosocial interventions (*Kandiah et al*).  **—****Physical activity interventions**: Physical activity may be recommended to adults (*WHO*); clinicians should recommend regular exercise (twice/week) as part of an overall approach to management (*Petersen et al*); The strategy should at least include physical exercise (*Kandiah et al*); We recommend physical activity interventions involving aerobic exercise to improve cognitive outcomes (*Ismail et al*).  **—****Cognitive interventions:** Clinicians may recommend cognitive interventions (*Petersen et al*); cognitive training can improve the overall cognitive function and functions of multiple cognitive domains (*Jia et al*), as well as reduce the risk of cognitive decline and/or dementia in healthy elderly and MCI patients (*WHO*); The strategy should at least include cognitive training (*Kandiah et al*).  **—Dietary and nutritional interventions:** Dietary and nutritional interventions should be considered alongside individualized lifestyle modifications (*Cummings et al*); the Mediterranean-like diet may be recommended to reduce the risk of cognitive decline and/or dementia (*WHO*); Souvenaid should be considered as a management option for patients with a diagnosis of MCI due to AD pathology (prodromal AD), and patients should take Souvenaid for 2 years or longer if there is evidence of continuing benefit; Souvenaid should be stopped if intolerance develops, the patient is no longer benefitting, or they progress to moderate-severe AD (*Cummings et al*); Wheat jujube porridge, Walnut Sesame Lotus Seed Porridge, Enhancing Wisdom and Kidney Cake, Ligustrum Jian (*Zhou et al*).  **—****Acupuncture:** For syndrome of blood stasis obstructing the collaterals: Neiguan (PC6) and Geshu (BL17). The positive syndrome acupuncture method is used to reduce the method, and the deficiency syndrome acupuncture method is used to replenish the method. Baihui (gv20) and Shenting (gv24) could also be chosen, and electroacupuncture could be performed (*Zhou et al*).  **Counseling:** Clinicians should discuss diagnosis and uncertainties regarding prognosis. Clinicians should counsel patients and families to discuss long-term planning topics such as advance directives, driving safety, finances, and estate planning (*Petersen et al*). | |

**Reference**

Brien, J.T.O., Jones, C.H.M., Livingston, G., Mittler, I.M.P., Ritchie, C., L, L.R.E., et al. (2017). Clinical practice with anti-dementia drugs: A revised (third) consensus statement from the British Association for Psychopharmacology. Journal of Psychopharmacology 31(2), 147-168.

Cummings, J., Passmore, P., McGuinness, B., Mok, V., Chen, C., Engelborghs, S., et al. (2019). Souvenaid in the management of mild cognitive impairment: An expert consensus opinion. Alzheimer's Research and Therapy 1(11), 73.

Dunne, R.A., Aarsland, D., O'Brien, J.T., Ballard, C., Banerjee, S., Fox, N.C., et al. (2021). Mild cognitive impairment: the Manchester consensus. AGE AND AGEING 50(1), 72-80.

Herukka, S.K., Simonsen, A.H., Andreasen, N., Baldeiras, I., Bjerke, M., Blennow, K., et al. (2017). Recommendations for cerebrospinal fluid Alzheimer's disease biomarkers in the diagnostic evaluation of mild cognitive impairment. Alzheimers Dement 13(3), 285-295. doi: 10.1016/j.jalz.2016.09.009.

Ismail, Z., and Richard, S.E.B. (2020). Recommendations of the 5th Canadian Consensus Conference on the diagnosis and treatment of dementia. Alzheimers Dement 16(8), 1182-1195.

Jia, J., Writing Group of Chinese Expert Consensus of Cognitive Training, and Chinese Medical Doctor Association Neurologist Branch Cognitive Disorders Professional Committee (2019). Chinese expert consensus of cognitive training. Natl Med J China 99(1), 4-8.

Jia, J., Writing group of Chinese guidelines for diagnosis and treatment of dementia and cognitive impairment, and Chinese Medical Doctor Association Neurologist Branch Cognitive Disorders Professional Committee (2018). Chinese guidelines for diagnosis and treatment of dementia and cognitive impairment in 2018 (five): diagnosis and treatment of mild cognitive impairment. Natl Med J China 17, 1294-1301.

Kandiah, N., and Christopher, Y.F.C. (2021). Strategies for the use of Ginkgo biloba extract, EGb 761 ® , in the treatment and management of mild cognitive impairment in Asia: Expert consensus. CNS NEUROSCI THER 27(2), 149-162.

Nobili, F., Arbizu, J., Bouwman, F., Drzezga, A., Agosta, F., Nestor, P., et al. (2018). European Association of Nuclear Medicine and European Academy of Neurology recommendations for the use of brain (18) F-fluorodeoxyglucose positron emission tomography in neurodegenerative cognitive impairment and dementia: Delphi consensus. Eur J Neurol 25(10), 1201-1217. doi: 10.1111/ene.13728.

Petersen, R.C., Lopez, O., Armstrong, M.J., Getchius, T.S.D., Mary Ganguli, D.G., Gary S Gronseth, D.M., et al. (2018). Practice guideline update summary: Mild cognitive impairment: Report of the Guideline Development, Dissemination, and Implementation Subcommittee of the American Academy of Neurology. Neurology 90(3), 126-135. doi: 10.1212/WNL.0000000000004826.

Tian, J., xie, H., Qin, B., Fan, D., Shi, J., and Wang, L. (2016). Chinese diagnostic guidelines of vascular mild cognitive impairment. Chin J Intern Med 3, 249-256.

WHO (2019). "Risk reduction of cognitive decline and dementia: WHO guidelines". (Geneva: World Health Organization).

Zhou, X., Huang, J., Xie, M., and Wu, C. (2020). Expert consensus of vascular mild cognitive impairment in Chinese traditional medicine. Chinese Journal of Information on Traditional Chinese Medicine 27(3), 1-5.
